# Cancer survival disparities by health insurance status

**DOI:** 10.1002/cam4.84

**Published:** 2013-05-08

**Authors:** Xiaoling Niu, Lisa M Roche, Karen S Pawlish, Kevin A Henry

**Affiliations:** 1 Cancer Epidemiology Services, New Jersey Department of HealthTrenton, New Jersey; 2 Department of Geography, University of UtahSalt Lake City, Utah; 3 Huntsman Cancer Institute, University of UtahSalt Lake City, Utah

**Keywords:** Bladder cancer, breast cancer, cervical cancer, colorectal cancer, disparities, insurance status, lung cancer, non-Hodgkin lymphoma, prostate cancer, survival

## Abstract

Previous studies found that uninsured and Medicaid insured cancer patients have poorer outcomes than cancer patients with private insurance. We examined the association between health insurance status and survival of New Jersey patients 18–64 diagnosed with seven common cancers during 1999–2004. Hazard ratios (HRs) with 95% confidence intervals for 5-year cause-specific survival were calculated from Cox proportional hazards regression models; health insurance status was the primary predictor with adjustment for other significant factors in univariate chi-square or Kaplan–Meier survival log-rank tests. Two diagnosis periods by health insurance status were compared using Kaplan–Meier survival log-rank tests. For breast, colorectal, lung, non-Hodgkin lymphoma (NHL), and prostate cancer, uninsured and Medicaid insured patients had significantly higher risks of death than privately insured patients. For bladder cancer, uninsured patients had a significantly higher risk of death than privately insured patients. Survival improved between the two diagnosis periods for privately insured patients with breast, colorectal, or lung cancer and NHL, for Medicaid insured patients with NHL, and not at all for uninsured patients. Survival from cancer appears to be related to a complex set of demographic and clinical factors of which insurance status is a part. While ensuring that everyone has adequate health insurance is an important step, additional measures must be taken to address cancer survival disparities.

## Background

Previous studies found that in the United States, uninsured and Medicaid insured patients with breast, cervical, colorectal, head and neck, lung, prostate or uterine cancer have higher mortality or lower survival than do patients with private insurance or Medicare, even after adjustment for other factors [1–13]. Authors of studies comparing cancer survival among Canadian residents with U.S. residents concluded that low-income Canadians have a survival advantage over low-income U.S. residents, probably due to Canada's universal health care system which provides equal access to medically necessary care [14, 15]. These and other studies also found that age, sex, race, ethnicity, socioeconomic status (SES), marital status, stage at diagnosis, comorbidities, behavioral risk factors, and treatment significantly impact survival from cancer [1–25]. As substantial proportions of the U.S. population are uninsured or enrolled in Medicaid – 48.6 million (15.7%) and 50.8 million (16.5%), respectively, in 2011 [26], it is important to determine differential effects of health insurance on health status.

We examined the association between health insurance status and cause-specific survival from seven common cancers diagnosed in New Jersey (NJ) residents aged 18–64 using a high-quality population-based cancer registry and adjusting for other significant factors. We excluded patients aged 65 or older because nearly all are insured through Medicare. We also compared cancer survival by insurance status between two time periods. The cancers we examined, female breast (breast), cervical, colorectal, lung and bronchus (lung), non-Hodgkin lymphoma (NHL), prostate and urinary bladder (bladder), accounted for 61% of the incident cancers and 56% of cancer deaths among NJ residents during 2005–2009 [27]. To our knowledge, this is the first study of survival disparities by insurance status to include NHL and urinary bladder cancer, as well as changes in the relationship between health insurance status and cancer survival over time.

## Methods

The New Jersey State Cancer Registry (NJSCR) is the population-based cancer incidence registry that serves the state of NJ, with a diverse population of over 8.7 million people. The NJSCR has participated in the Centers for Disease Control and Prevention's National Program of Cancer Registries since it began and is a National Cancer Institute (NCI) Surveillance, Epidemiology and End Results (SEER) expansion registry. The NJSCR includes patient demographics and clinical information (e.g., date of diagnosis, stage at diagnosis, primary payer at diagnosis, or first course of treatment) on each cancer case. The primary site and histology of each case are coded to the International Classification of Diseases for Oncology (ICD-O), 3rd edition [28] and the stage at diagnosis is coded according to SEER summary stage [29, 30]. The North American Association of Central Cancer Registries awarded the Gold Standard to the NJSCR for quality and completeness of 1995 through 2009 data. Additional details of NJSCR operations are in the most recent annual report [27].

All first primary invasive breast, cervical, colorectal, lung, prostate, and bladder (also *in situ*) cancers and NHLs in the NJSCR diagnosed during 1999–2004 in persons aged 18–64 years were included. The ICD-O-3 codes for the seven cancers are those in the SEER site recode definition [31]. Cases were excluded if: ascertained by death certificate or autopsy report only; health insurance status other than private, Medicaid or uninsured, for example, Medicare, military, Indian Health Service; race other than white, black, or Asian/Pacific Islander (API); unknown race or insurance status or no survival time.

Vital status in the NJSCR is updated annually through linkages with state and national death files, state taxation files, hospital discharge files, Medicare and Medicaid files, Social Security Administration Services for Epidemiologic Researchers and motor vehicle registration files. Additionally, hospitals are required to submit annual vital status updates on all cases they have reported. Completeness of vital status follow-up in December 2011 (when the study data file was prepared) for the 54,002 study cases was 97%, ranging from 92% for cervical cancer to 99% for lung cancer. Cause of death codes were obtained from the state and national death file in the NJSCR.

After linkage with NJ hospital discharge data using Link Plus (CDC software), 6.1% of the eligible cases (4.3% to 9.4% depending on cancer type) had unknown primary payer compared with 8.3% before the linkage. About 6% of the cases had been uninsured as the primary payer after the linkage versus 7% before the linkage.

### Data analysis

Five-year cause-specific survival, the measure of cancer survival used in this study, is the probability of surviving a specific cause of death in the absence of other causes of death. Survival time in months for each case was calculated from the date of diagnosis to the date of death from any cancer or to 5 years after diagnosis if known to be alive then. Cases whose cause of death was not cancer or who were lost to follow-up were censored at that time. For each cancer type, associations between health insurance status and age, sex, race/ethnicity, census tract SES based on a deprivation index described below, marital status, and stage were assessed with chi-square tests.

Kaplan–Meier 5-year cause-specific survival curves with log-rank statistical significance tests were calculated for each cancer type by the above-listed variables as well as by insurance status. Hazard ratios (HRs) with 95% confidence intervals (CIs) for cause-specific survival within 5 years were calculated from Cox proportional hazards regression models; health insurance status was the primary predictor with adjustment for other statistically significant variables in the chi-square or Kaplan–Meier survival log-rank tests. The proportional hazards assumption was confirmed from the Kaplan–Meier survival curves for Medicaid insured and uninsured compared with privately insured patients [32]. To ascertain change in survival over time, Kaplan–Meier 5-year cause-specific survival curves with log-rank statistical significance tests by two diagnosis periods (1999–2001, 2002–2004) for each health insurance status were calculated. *P*-values <0.05 were considered statistically significant and *P*-values ≥0.05 but <0.10 were of borderline statistical significance.

Health insurance status based on primary payer was categorized as private, Medicaid, or uninsured; for the HRs, the private insurance status category was the referent. Age was categorized as: 18–39, 40–54, 55–64 except as 18–54 and 55–64 for prostate cancer due to very small numbers in the 18–39 age group; race/ethnicity as non-Hispanic white, non-Hispanic black, non-Hispanic API, Hispanic; marital status as married, not married (single, separated, divorced, widowed, unknown), and stage as SEER summary stage local, regional, distant, or unknown. SAS version 9.2 (SAS Institute Inc., Cary, NC) was used for all analyses.

As the NJSCR does not collect individual SES information, census tract SES measures from the U.S. Census for NJ were used to develop a standardized deprivation index using principle component analysis [33], as described in a previous article [34]. NJ census tracts were grouped into SES quartiles based on their deprivation index scores; the higher the deprivation index score, the more deprived the tract. Cases were categorized into SES quartiles according to their geocoded census tract. For the HRs, the highest SES quartile was the referent.

## Results

After exclusions, 54,002 cases remained of the 63,429 eligible cases; among the cases excluded were 217 cases ascertained by death certificate or autopsy report only and 8363 cases with Medicare, military, or unknown health insurance status. For each nonsex-specific cancer type, males represented 53% to 75% of the cases and the distribution of cases by age, race, ethnicity, marital status, and SES varied greatly (Table 1). The proportion of cases diagnosed at the distant stage was between 2% (prostate, bladder) and 54% (lung). The percentage of uninsured cases ranged from 5% (breast, prostate, bladder) to 18% (cervical) and the percentage with Medicaid ranged from 2% (prostate) to 9% (cervical). Women with cervical cancer also were more likely to be young (32%), Hispanic (20%), in the lowest SES quartile (33%), and not married (53%) than patients with other types of cancer.

**Table 1:** Demographics, stage at diagnosis, and health insurance status by cancer type, New Jersey, 1999–2004, *N* = 54,002

	Breast (*n* = 17,939), *n* (%)	Cervical (*n* = 1,832), *n* (%)	Colorectal (*n* = 7,445), *n* (%)	Lung (*n* = 8,185), *n* (%)	NHL^1^ (*n* = 3,885), *n* (%)	Prostate (*n* = 11,842), *n* (%)	Bladder (*n* = 2,874), *n* (%)
Sex							
Male	–	–	4,201 (56.4)	4,359 (53.3)	2,228 (57.3)	11,842 (100)	2,168 (75.4)
Female	17,939 (100)	1,832 (100)	3,244 (43.6)	3,826 (46.7)	1,657 (42.7)	–	706 (24.6)
Age							
18–39	1,927 (10.7)	582 (31.7)	563 (7.5)	226 (2.7)	712 (18.3)	20 (0.2)	123 (4.2)
40–54	9,575 (53.4)	900 (49.1)	2,955 (39.7)	2,884 (35.2)	1,700 (43.8)	3,039 (25.7)	1,030 (35.8)
55–64	6,438 (35.9)	351 (19.2)	3,928 (52.8)	5,076 (62.0)	1,474 (37.9)	8,784 (74.2)	1,722 (59.9)
Race/Ethnicity							
NH^2^ white	13,524 (75.4)	1,085 (59.2)	5,279 (70.9)	6,368 (77.8)	2,811 (72.4)	8,607 (72.7)	2,514 (87.5)
NH black	1,998 (11.1)	319 (17.4)	1,121 (15.1)	1,168 (14.3)	505 (13.0)	2,044 (17.3)	144 (5.0)
NH API^3^	838 (4.7)	64 (3.5)	332 (4.5)	202 (2.5)	146 (3.8)	241 (2.0)	63 (2.2)
Hispanic	1,579 (8.8)	364 (19.9)	713 (9.6)	447 (5.5)	423 (10.9)	950 (8.0)	153 (5.3)
Marital status							
Married	11,802 (65.8)	866 (47.3)	5,020 (67.4)	4,870 (59.5)	2,435 (62.7)	9,026 (76.2)	2,055 (71.5)
Not married^4^	6,137 (34.2)	966 (52.7)	2,425 (32.6)	3,315 (40.5)	1,450 (37.3)	2,816 (23.8)	819 (28.5)
SES quartile^5^							
Quartile 1	6,366 (35.5)	362 (19.8)	2,137 (28.7)	1,826 (22.3)	1,191 (30.7)	4,302 (36.3)	939 (32.7)
Quartile 2	4,962 (27.7)	407 (22.2)	2,054 (27.6)	2,294 (28.0)	1,059 (27.3)	3,104 (26.2)	862 (30.0)
Quartile 3	3,917 (21.8)	452 (24.7)	1,815 (24.4)	2,315 (28.3)	881 (22.7)	2,483 (21.0)	708 (24.6)
Quartile 4	2,694 (15.0)	611 (33.4)	1,439 (19.3)	1,750 (21.4)	754 (19.4)	1,953 (16.5)	365 (12.7)
Stage^6^							
Local	10,107 (56.3)	920 (50.2)	2,480 (33.3)	1,128 (13.8)	1,172 (30.2)	9,903 (83.6)	2,526 (87.9)
Regional	6,617 (36.9)	630 (34.4)	3,066 (41.2)	2,244 (27.4)	600 (15.4)	1,397 (11.8)	183 (6.4)
Distant	846 (4.7)	175 (9.6)	1,574 (21.1)	4,380 (53.5)	1,711 (44.0)	265 (2.2)	65 (2.3)
Unknown	369 (2.1)	107 (5.8)	325 (4.4)	433 (5.3)	402 (10.3)	277 (2.3)	100 (3.5)
Insurance							
Uninsured	967 (5.4)	320 (17.5)	578 (7.8)	822 (10.0)	299 (7.7)	590 (5.0)	150 (5.2)
Medicaid	591 (3.3)	167 (9.1)	300 (4.0)	557 (6.8)	210 (5.4)	199 (1.7)	76 (2.6)
Private	16,381 (91.3)	1,345 (73.4)	6,567 (88.2)	6,806 (83.2)	3,376 (86.9)	11,053 (93.3)	2,648 (92.1)
Diagnosis period							
1999–2001	9,023 (50.3)	921 (50.3)	3,659 (49.1)	4,137 (50.5)	1,966 (50.6)	5,835 (49.3)	1,419 (49.4)
2002–2004	8,916 (49.7)	911 (49.7)	3,786 (50.9)	4,048 (49.5)	1,919 (49.4)	6,007 (50.7)	1,455 (50.6)

1 Non-Hodgkin lymphoma.

2 Non-Hispanic.

3 Asian/Pacific Islander.

4 Includes single, separated, divorced, widowed, and unknown marital status.

5 Highest SES quartile is quartile 1, lowest SES quartile is quartile 4.

6 Stage at diagnosis, local stage includes *in situ* for bladder cancer.

Significant sex differences in insurance status occurred only for lung cancer, with higher proportions of men than women uninsured or Medicaid insured (*P* < 0.0001). Age differences in insurance status were statistically significant for breast, colorectal and lung cancer and NHL (*P* ≤ 0.0002), with the youngest age group most likely uninsured or Medicaid insured. Within each cancer type, Hispanics were most likely uninsured and non-Hispanic blacks most likely had Medicaid compared with the other race/ethnic groups, except Hispanic colorectal and bladder cancer cases most likely had Medicaid (*P* < 0.0001). Unmarried cases with each type of cancer were far more likely uninsured or Medicaid insured than married cases (*P* < 0.0001). Across all cancer types, much higher percentages of the lowest SES quartile cases were uninsured or had Medicaid, with higher percentages of uninsured or Medicaid cases in each successively lower SES quartile (*P* < 0.0001). Cases diagnosed at the distant or unknown stage were more likely uninsured or Medicaid insured than cases diagnosed at the local or regional stage (*P* ≤ 0.0015).

### Kaplan–Meier cause-specific 5-year survival

**Figure 1:**
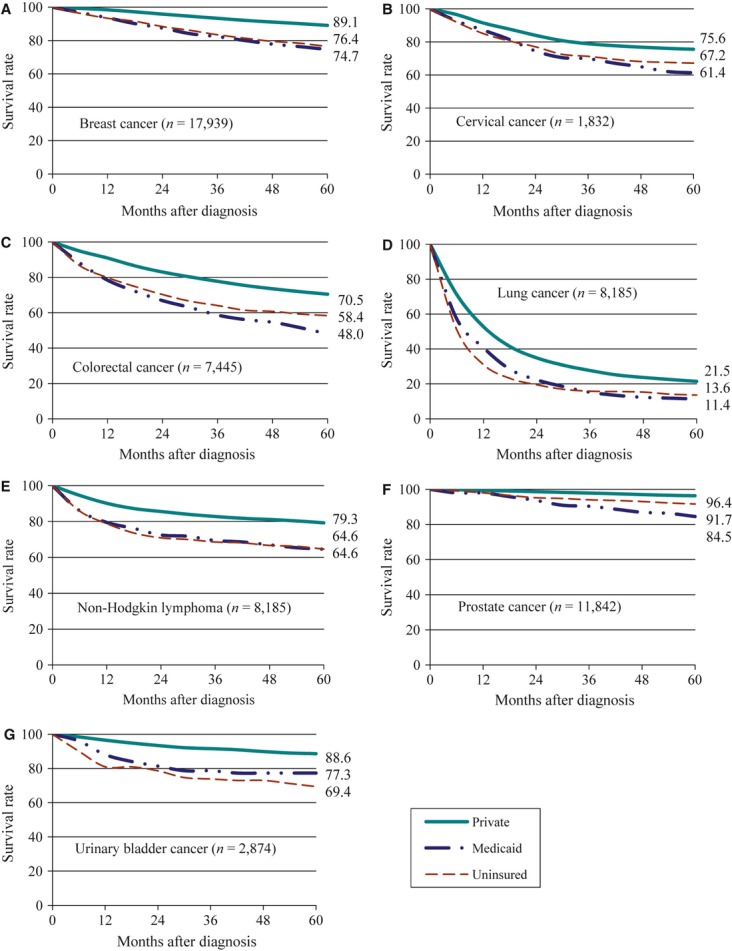
Five-year cause-specific survival rates by health insurance status for each cancer type, New Jersey, 1999–2004. The rates were significantly different by insurance status for each cancer type (Kaplan–Meier log-rank tests, *P* < 0.0001). The total numbers of cancers and cause-specific 5-year rates are labeled in each figure.

Estimated 5-year cause-specific survival was highest for prostate cancer (96.0%) and lowest for lung cancer (20.0%), with intermediate rates for breast (88.0%), bladder (87.4%), NHL (77.6%), cervical (73.0%), and colorectal (68.7%) cancer. For each cancer, uninsured and Medicaid insured patients had statistically significantly lower survival rates than privately insured patients; 5 to 19 and 10 to 22 percentage points lower, respectively, than the analogous privately insured patients' rates (Fig. 1). The survival difference between Medicaid insured and uninsured patients was not statistically significant for each of the seven cancers.

Women had a survival advantage over men for colorectal cancer, lung cancer, and NHL (*P* < 0.05) while men had a survival advantage for bladder cancer although of borderline statistical significance (*P* = 0.06). The youngest cervical, lung, prostate, or bladder cancer patients and the middle age group of breast cancer and NHL patients had the highest survival rates. There were significant racial/ethnic survival disparities for each cancer type (*P* < 0.0001) except cervical, with lowest survival among non-Hispanic blacks and next lowest survival among Hispanics. For every cancer type, the lower the SES quartile the lower survival (*P* < 0.0001) and unmarried patients had lower survival than married patients (*P* < 0.0001). Survival rates for patients diagnosed at the distant stage were by far the lowest while patients diagnosed at the local stage had the greatest survival (*P* < 0.0001).

### Cox regression models and HRs

After adjustment for factors that were statistically significantly associated with survival (Kaplan–Meier survival log-rank tests) and/or insurance status (chi-square tests), uninsured patients had significantly higher risks of death within 5 years of diagnosis than privately insured patients for breast, colorectal, lung, NHL, prostate, and bladder cancers (HRs = 1.44, 1.41, 1.43, 1.69, 1.97, 1.76, respectively, Table 2). Similarly, Medicaid insured patients had significantly higher risks of death within 5 years than privately insured patients for female breast, colorectal, lung, NHL, and prostate cancer (HRs = 1.56, 1.57, 1.21, 1.48, 2.98) and a nonsignificant higher risk for death from bladder cancer (HR = 1.37). For cervical cancer, uninsured patients had the same risk of death within 5 years (HR = 1.00) while Medicaid insured patients had a nonsignificant higher risk of death (HR = 1.32) compared with privately insured patients.

**Table 2:** Hazard ratios and 95% confidence intervals within 5 years of cancer diagnosis by health insurance status, New Jersey, 1999–2004, *N* = 54,002

	Health insurance status^1^		
	
Cancer type	Medicaid HR (95% CI)	Uninsured HR (95% CI)	Private referent
Breast (*n* = 17,939)	1.56 (1.29–1.88)	1.44 (1.22–1.69)	1
Cervical (*n* = 1832)	1.32 (0.94–1.86)	1.00 (0.75–1.34)	1
Colorectal (*n* = 7445)	1.57 (1.28–1.93)	1.41 (1.20–1.66)	1
Lung (*n* = 8185)	1.21 (1.08–1.35)	1.43 (1.31–1.57)	1
NHL^2^ (*n* = 3885)	1.48 (1.04–2.10)	1.69 (1.29–2.23)	1
Prostate (*n* = 11,842)	2.98 (1.92–4.64)	1.97 (1.41–2.77)	1
Bladder (*n* = 2874)	1.37 (0.72–2.63)	1.76 (1.14–2.71)	1

1 Hazard ratios (HR) and 95% confidence intervals (95% CI) are from Cox proportional hazards regression models for cause-specific survival within 5 years of diagnosis as follows. Breast, cervical, prostate, and urinary bladder cancers adjusted for age, race/ethnicity, SES, marital status, and stage. Colorectal and lung cancers and non-Hodgkin lymphoma adjusted for the same variables plus sex.

2 Non-Hodgkin lymphoma.

### Comparison of two time periods

Five-year survival improved between the 1999–2001 and 2002–2004 diagnosis periods for privately insured patients with breast cancer (*P* = 0.05), colorectal cancer (*P* = 0.02), lung cancer (*P* = 0.06), and NHL (*P* = 0.001) by 1 to 5 percentage points, worsened for cervical cancer (*P* = 0.09) by 4 percentage points and did not significantly change for prostate or bladder cancer. Uninsured patients' survival did not significantly improve or worsen; however, for Medicaid insured NHL patients 5-year survival improved (*P* = 0.03) by 16 percentage points (Table 3).

**Table 3:** Five-year cause-specific survival rates by health insurance status and cancer type for two diagnosis periods, 1999–2001 and 2002–2004, New Jersey, *N* = 54,002

	Health insurance status^1^						
	
	Medicaid		Uninsured		Private	
			
Cancer type	1999–2001	2002–2004	1999–2001	2002–2004	1999–2001	2002–2004
Breast	74.6%	74.7%	75.7%	77.4%	88.6%	89.6%
Cervix	62.2%	60.6%	65.9%	68.3%	77.6%	73.6%
Colorectal	46.4%	49.5%	55.8%	60.7%	69.2%	71.8%
Lung	11.5%	11.3%	13.8%	13.4%	20.4%	22.6%
NHL^2^	57.5%	73.3%	64.3%	65.1%	76.9%	81.7%
Prostate	84.6%	84.6%	91.4%	92.0%	96.3%	96.5%
Bladder	80.1%	74.2%	70.1%	68.7%	87.7%	89.5%

1 Five-year cause-specific survival rates were calculated using Kaplan–Meier method.

2 Non-Hodgkin lymphoma.

## Discussion

Among NJ patients 18–64 years old with breast, colorectal, lung, prostate, bladder cancer, or NHL, those without insurance had a significantly higher risk of death within 5 years of diagnosis (41%–97%) than those with private insurance even after adjustment for important prognostic factors such as gender, age, race/ethnicity, marital status, SES, and stage. Medicaid insured patients with these same cancers (except bladder cancer) also had significantly higher risks of dying within 5 years of diagnosis than those with private insurance – 21% to 198%. Our results for breast, colorectal, lung, and prostate cancer are comparable to previous studies' results, although the populations and years studied and analytic methods were different [2, 4, 5, 7, 9–13]. The previous study of NJ breast cancer patients diagnosed in 1985–1987 found 1.49 and 1.40 adjusted relative risks of death among uninsured and Medicaid insured women, respectively, compared with privately insured women [13], similar to our adjusted HRs of 1.44 and 1.56 for breast cancer patients diagnosed in 1999–2004. Thus, among NJ women with breast cancer, survival disparities by insurance status appear not to have changed in the past several decades.

Possible reasons for uninsured and Medicaid insured cancer patients' poorer survival compared with privately insured cancer patients, even after adjustment for other factors, may include: poorer health with more comorbidity and unhealthy behaviors; no or inadequate preventive health care and management of chronic conditions prior to cancer diagnosis; barriers to receiving treatment and adhering to a treatment regimen such as high cost, inability to navigate the health care system, misinformation about and mistrust of the health care system, lack of a usual source of health care, lack of transportation, lack of time off from work; no treatment or delay in receiving treatment; not all providers accept uninsured or Medicaid insured patients; and lower quality treatment by providers primarily serving the uninsured and Medicaid insured [3, 4, 8, 9, 12].

A recent study found that patients insured through Medicaid after cancer diagnosis had higher disease-specific mortality than patients insured through Medicaid before cancer diagnosis and that both Medicaid insured groups had significantly higher mortality than the non-Medicaid insured group [35]. The authors noted that in other studies cancer patients enrolled in Medicaid before diagnosis compared to cancer patients enrolled after diagnosis were more likely to receive screening mammography and be diagnosed at earlier stages [35].

For cervical cancer, we found no significant difference in survival between uninsured or Medicaid insured versus privately insured patients when other factors were taken into account, similar to results from a previous study of cervical cancer survival in Florida [6]. Authors of the Florida study concluded that racial, ethnic, and SES disparities in cervical cancer survival were explained by late-stage presentation and undertreatment [6]. NJ cervical cancer patients appear to be particularly vulnerable with relatively high proportions in demographic groups with poorer survival generally, that is, non-Hispanic black, Hispanic, low SES, unmarried, without insurance or Medicaid insured, than patients with other types of cancer (Table 1). As cervical cancer is mostly preventable with human papillomavirus vaccination (HPV) and Pap tests (which detect precancerous lesions), emphasis needs to remain on reaching all women with these measures.

The results from the comparison of two time periods showed that while 5-year survival significantly improved or remained the same for privately insured patients (except those with cervical cancer), survival did not improve for uninsured or Medicaid insured patients (except Medicaid insured patients with NHL). Thus, the survival disparities between privately insured and uninsured or Medicaid insured patients widened over time. The much greater improvement in survival over time for Medicaid insured NHL patients was unexpected and cannot be explained by this study.

We found no other studies with which to compare our study results relating to NHL and bladder cancer survival, or changes over time in the relationship between insurance status and cancer survival.

### Limitations

Our results could be out of date since we did not use the most recent years of NJSCR data, diagnosis years 2005–2009, in order to allow 5 years of follow-up for each case. However, our comparison of survival between two time periods showed improvement primarily for the insured patients and little or no improvement for Medicaid insured (except NHL patients) and uninsured patients. If this trend continued beyond the 2004 diagnosis year then survival disparities between uninsured and Medicaid insured patients versus privately insured patients would be expected to have increased. Some patients' insurance status may have been misclassified, despite the NJSCR and NJ hospital discharge data linkage, due to errors in medical records, changes in insurance between cancer diagnosis and treatment, etc.

Using census tract level SES may result in misclassification of cases with higher or lower SES than their census tract. However, previous research indicates that census tract level SES measures substitute well for individual measures of SES [36]. Also, some misclassification of cases' SES due to changes between 2000 and 2004 in the variables used in the deprivation index and to census tract geocoding errors likely occurred.

We were unable to include some factors known to affect survival such as treatment regimen, comorbidities, and risky behavior. Previous studies found survival disparities between uninsured and Medicaid insured patients versus privately insured patients with these factors taken into account [1–3, 5, 7, 9, 11, 13].

Another possible study limitation is that we calculated cause-specific survival because we could not calculate relative survival due to lack of New Jersey-specific life tables. An underlying assumption in cause-specific survival is that the cause of death on death certificates is accurate. Howlader et al. [37], based on a comparison of 5-year cause-specific survival rates with relative survival rates using SEER data, concluded that cause-specific survival may be a viable alternative to relative survival when appropriate life tables are not available. Also, as mentioned above, where we could compare, our results are similar to those of previous studies in which relative survival was calculated.

This study involved multiple statistical tests so false positives could have occurred; however, the very low *P*-values for many of the significant results provide a measure of confidence. It is also possible that lead-time bias could explain better survival for privately insured patients, especially for the cancers with population-based screening methods (breast, cervical, colorectal, prostate). However, the inclusion of stage at diagnosis in the analyses may have mitigated this problem. Regarding the time trend analysis, we were not able to evaluate longer term trends in survival due to incomplete information on insurance in the NJSCR for cases diagnosed before 1999.

## Conclusions

Survival from cancer appears to be related to a complex set of interrelated demographic and clinical factors of which insurance status is a part. The finding that Medicaid insured cancer patients also have worse survival than privately insured cancer patients suggests that while ensuring that everyone has adequate health insurance is an important step, additional measures are needed to address cancer survival disparities. These include: building capacity in the U.S. public health and health care systems, especially in underserved communities; education about cancer prevention, detection, and treatment; preventive and chronic health care before a diagnosis of cancer; assistance to cancer patients in accessing and navigating the health care system; and workplace policies that encourage patients' attention to their health.

## References

[1] Fedewa SA, Lerro C, Chase D, Ward EM (2011). Insurance status and racial differences in uterine cancer survival: a study of patients in the National Cancer Database. Gynecol. Oncol.

[2] Robbins AS, Chen AY, Stewart AK, Staley CA, Virgo KS, Ward EM (2010). Insurance status and survival disparities among nonelderly rectal cancer patients in the National Cancer Data Base. Cancer.

[3] Kwok J, Langevin SM, Argiris A, Grandis JR, Gooding WE, Taioli E (2010). The impact of health insurance status on the survival of patients with head and neck cancer. Cancer.

[4] Slatore CG, Au DH, Gould MK (2010). An official American Thoracic Society systematic review: insurance status and disparities in lung cancer practices and outcomes. Am. J. Respir. Crit. Care Med.

[5] Robbins AS, Pavluck AL, Fedewa SA, Chen AY, Ward EM (2009). Insurance status, comorbidity level, and survival among colorectal cancer patients age 18 to 64 years in the National Cancer Data Base from 2003 to 2005. J. Clin. Oncol.

[6] Brookfield KF, Cheung MC, Lucci J, Fleming LE, Koniaris LG (2009). Disparities in survival among women with invasive cervical cancer. Cancer.

[7] Cheung MC, Hamilton K, Sherman R, Byrne MM, Nguyen DM, Franceschi D (2009). Impact of teaching facility status and high-volume centers on outcomes for lung cancer resection: an examination of 13,469 surgical patients. Ann. Surg. Oncol.

[8] Ward E, Halpern M, Schrag N, Cokkinides V, DeSantis C, Bandi P (2008). Association of insurance with cancer care utilization and outcomes. CA Cancer J. Clin.

[9] McDavid K, Tucker TC, Sloggett A, Coleman MP (2003). Cancer survival in Kentucky and health insurance coverage. Arch. Intern. Med.

[10] Roetzheim RG, Gonzalez EC, Ferrante JM, Pal N, Krischer DJ, Van Durme JP (2000). Effects of health insurance and race on breast carcinoma treatments and outcomes. Cancer.

[11] Roetzheim RG, Pal N, Gonzalez EC, Ferrante JM, Krischer DJ, Van Durme JP (2000). Effects of health insurance and race on colorectal cancer treatments and outcomes. Am. J. Public Health.

[12] Lee-Feldstein A, Feldstein PJ, Buchmueller T, Katterhagen G (2000). The relationship of HMOs, health insurance, and delivery systems to breast cancer outcomes. Med. Care.

[13] Ayanian JZ, Kohler BA, Abe T, Epstein AM (1993). The relation between health insurance coverage and clinical outcomes among women with breast cancer. N. Engl. J. Med.

[14] Gorey KM, Luginaah IN, Bartfay E, Fung KY, Holowaty EJ, Wright FC (2011). Effects of socio-economic status on colon cancer treatment accessibility and survival in Toronto, Ontario, and San Francisco, California, 1996–2006. Am. J. Public Health.

[15] Gorey KM (2009). Breast cancer survival in Canada and the USA: meta-analytic evidence of a Canadian advantage in low-income areas. Int. J. Epidemiol.

[16] Robbins AS, Siegel RL, Jemal A (2012). Racial disparities in stage-specific colorectal cancer mortality rates from 1985–2009. J. Clin. Oncol.

[17] Menashe I, Anderson WF, Jatoi I, Rosenberg PS (2009). Underlying causes of the black-white racial disparity in breast cancer mortality: a population-based analysis. J. Natl. Cancer Inst.

[18] Scosyrev E, Noyes K, Feng C, Messing E (2009). Sex and racial differences in bladder cancer presentation and mortality in the US. Cancer.

[19] Brookfield KF, Cheung MC, Gomez C, Yang R, Nieder AM, Lee DJ (2009). Survival disparities among African American women with invasive bladder cancer in Florida. Cancer.

[20] Datta GD, Neville BA, Kawachi I, Datta NS, Earle CC (2009). Marital status and survival following bladder cancer. J. Epidemiol. Community Health.

[21] Ou SH, Zell JA, Ziogas A, Anton-Culver H (2008). Low socioeconomic status is a poor prognostic factor for survival in stage I nonsmall cell lung cancer and is independent of surgical treatment, race, and marital status. Cancer.

[22] Wang M, Burau KD, Fang S, Wang H, Du XL (2008). Ethnic variations in diagnosis, treatment, socioeconomic status, and survival in a large population-based cohort of elderly patients with non-Hodgkin lymphoma. Cancer.

[23] Robbins AS, Yin D, Parikh-Patel A (2007). Differences in prognostic factors and survival among white men and black men with prostate cancer, California, 1995–2004. Am. J. Epidemiol.

[24] Niu X, Pawlish K, Roche LM (2010). Cancer survival disparities by race/ethnicity and socioeconomic status in New Jersey. J. Health Care Poor Underserved.

[25] Henry KA, Niu X, Boscoe FP (2009). Geographic disparities in colorectal cancer survival. Int. J. Health Geogr.

[26] U.S. Census Bureau (2012). http://www.census.gov/prod/2012pubs/p60-243.pdf.

[27] Niu X, Burger S, Pawlish K, Graff J (2012). Cancer incidence and mortality in New Jersey, 2005–2009.

[28] Fritz A, Percy C, Jack A, Shanmugaratnam K, Sobin L, Parkin DM (2000). International classification of diseases for oncology.

[29] Young JL, Roffers SD, Ries LAG, Fritz AG, Hurlbut AA (2001). SEER summary staging manual – 2000: codes and coding instructions, NIH publication number 01–4969.

[30] Adamo MB, Johnson CH, Ruhl JL, Dickie LA (2010). SEER program coding and staging manual.

[31] SEER Site Recode ICD-O-3 (2003). http://seer.cancer.gov/siterecode/icdo3-d01272003/(accessed.

[32] Cantor AB (2003). SAS survival analysis techniques for medical research.

[33] Messer LC, Laraia BA, Kaufman JS, Eyster J, Holzman C, Culhane J (2006). The development of a standardized neighborhood deprivation index. J. Urban Health.

[34] Roche LM, Niu X, Pawlish KS, Henry KA (2011). Thyroid cancer incidence in New Jersey: time trend, birth cohort and socioeconomic status analysis 1979–2006. J. Environ. Public Health.

[35] Koroukian SM, Bakaki PM, Raghavan D (2012). Survival disparities by Medicaid status: an analysis of 8 cancers. Cancer.

[36] Krieger N, Chen JT, Waterman PD, Rehkopf DH, Subramanian SV (2005). Painting a truer picture of socioeconomic and racial/ethnic inequalities: the public health disparities geocoding project. Am. J. Public Health.

[37] Howlader N, Ries LA, Mariotto AB, Reichman ME, Ruhl J, Cronin KA (2010). Improved estimates of cancer-specific survival rates from population-based data. J. Natl. Cancer Inst.

